# Supplementary material for: Evolutionary modification of AGS protein contributes to formation of micromeres in sea urchins
Source: Nat Commun. 2019 Aug 22;10:3779. doi: 10.1038/s41467-019-11560-8 (PMC6706577; doi:10.1038/s41467-019-11560-8)
Supplement: Supplementary file 1 — Supplementary information [file 41467_2019_11560_MOESM1_ESM.pdf]

Supplementary Information

Evolutionary modification of AGS protein contributes to formation of micromeres in sea urchins

Poon et al.

Outlines:

Supplementary Figure 1. Asymmetric localization of molecules (cell fate factors) during two successive asymmetric cell divisions.

Supplementary Figure 2. Cortical AGS and Gαi localizes to the vegetal pole during asymmetric cell divisions.

Supplementary Figure 3. AGS-MO kd causes defects in micromere formation and endoderm specification.

Supplementary Figure 4. Control IP and Immunoblot results

Supplementary Figure 5. Localization and function of PLK1 during embryogenesis.

Supplementary Figure 6. AGS co-localizes with microtubules at the cortex in AGS-overexpressing embryos both in live and fixed samples.

Supplementary Figure 7. Sea urchin AGS (SpAGS) induces asymmetric cell divisions, and the lineage tracing of the micromere-like cells in the sea star embryos.

Supplementary Table 1. Summary of molecules described in this study.

Supplementary Table 2. SpAGS Blast with Human LGN and AGS3.

Supplementary Table 3. Alignment of SpGαi with Human Gai.

Supplementary Table 4. SpPLK1 alignment with Human PLK1.

Supplementary Table 5. Sea urchin SpAGS alignment with Sea star PmAGS.

Supplementary Table 6. Alignment of echinoderm AGS proteins.

Supplementary Table 7. Motif prediction of echinoderm AGS proteins (corresponding to Fig. 9a).

Supplementary Table 8. A key resource table.

Supplementary References

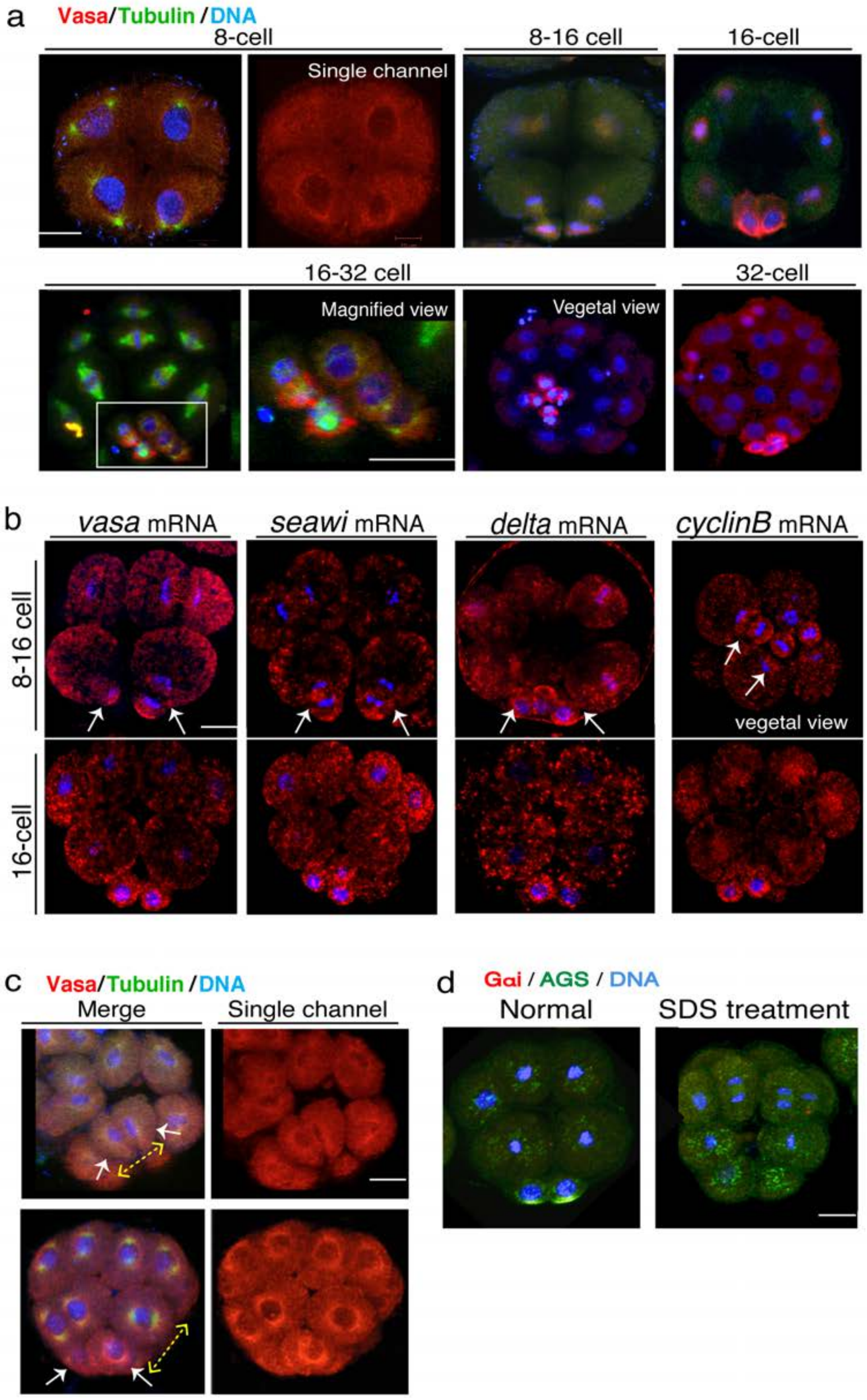

Supplementary Figure 1. Asymmetric localization of molecules (cell fate factors) during two successive asymmetric cell divisions. **a,** Asymmetric localization of Vasa during two successive asymmetric cell divisions. Vasa (red) is localized to the Vegetal side (micromere side) prior to cytokinesis at 8-cell and its asymmetric localization is retained during the formation of small micromeres at the 16-32 cell stage. Vasa, red; Tubulin, green; DNA; blue. **b,** Asymmetric localization of mRNAs (*vasa, seawi, delta, cyclinB*) over the micromere-side of the spindle (arrows) during asymmetric cell division was visualized by fluorescent *in situ* hybridization (red). DNA, blue. **c & d,** SDS treatment at mid 8-cell stage induced two types of equal-sized cell divisions at 16-cell stage. (c) In the top panels, the orientation of the spindle was rotated by 90° and became horizontal like in the mesomeres (61%, n= 92). In the bottom panels, the spindle did not change the orientation yet the embryo formed macromeres and micromeres that are equal in size (18%, n=92). In either of the cases, disruption of asymmetric cell division inhibited Vasa accumulation in the micromere-like cells (arrows). Dashed lines indicate the orientation of cell division. Vasa, red; Tubulin, green; DNA, blue. (d) Cortical AGS/Gαi signal at the vegetal cortex was lost in the embryos perturbed with unequal cell division at 16-cell stage. Gαi, red; AGS, green; DNA, blue. Unless individually indicated, the representative phenotypes of 70% or larger in each population are shown (n > 20). Scale bars = 20μm.

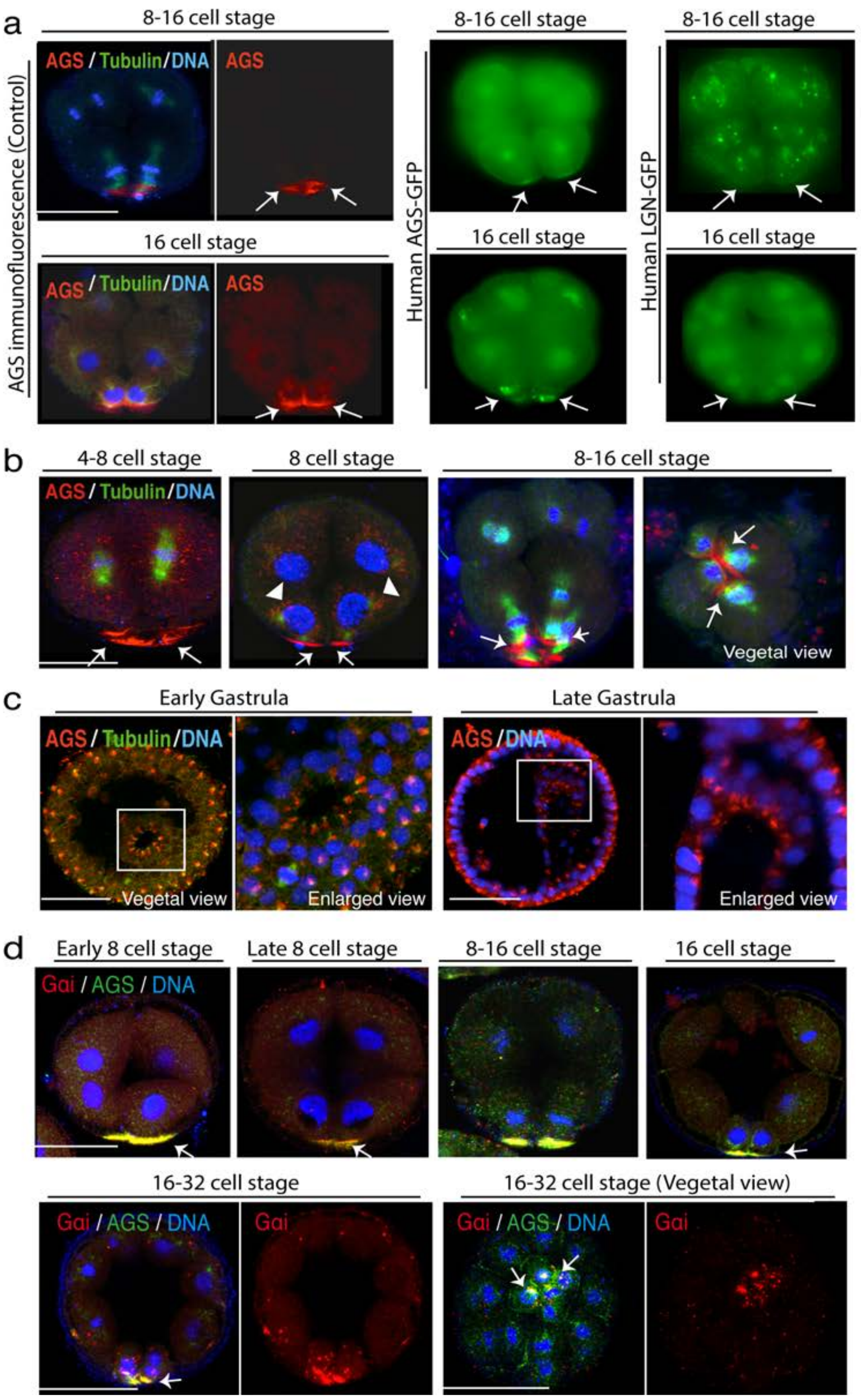

Supplementary Figure 2. Cortical AGS and Gαi localizes to the vegetal pole during asymmetric cell divisions. **a,** Left panels: immunofluorescence images showing endogenous AGS (red) localization at the cortex (arrows) at 8-16 cell or 16-cell stage as a control. Right panels: Human AGS-GFP demonstrated its localization at the cortex (arrows) besides on the centrosomes/spindle, whereas Human LGN-GFP signal was absent from the cortex (arrows). Human AGS-GFP or LGN-GFP mRNA (1 μg/μl stock) was injected into fertilized eggs of the sea urchin. **b,** Immunofluorescence image of 8-16 cell stage embryos. Cytoplasmic AGS is present on the mitotic apparatus of every blastomere (arrowheads). Cortical AGS (red) is, on the other hand, specifically localized at the vegetal pole from the 4-8 cell stage and during asymmetric cell divisions (arrows), suggesting that a single AGS may function in both cortical and spindle roles. Tubulin, green; DNA, blue. **c,** Immunofluorescence image of gastrula embryos (Day2 PF). AGS (red) is localized on the centrosomes of every cell yet no cortical localization was detected. Each of white windows indicates a region magnified in the right. Tubulin, green, DNA, blue. **d,** Gαi (red) is specifically localized at the vegetal pole from 8-cell stage and during asymmetric cell divisions (arrows). Unlike AGS, cytoplasmic Gαi was not detected and its localization was strictly restricted at the tip of the vegetal pole. AGS, green; DNA, blue. The representative phenotypes of 85% or larger in each population are shown ($n > 30$). Scale bars =50μm.

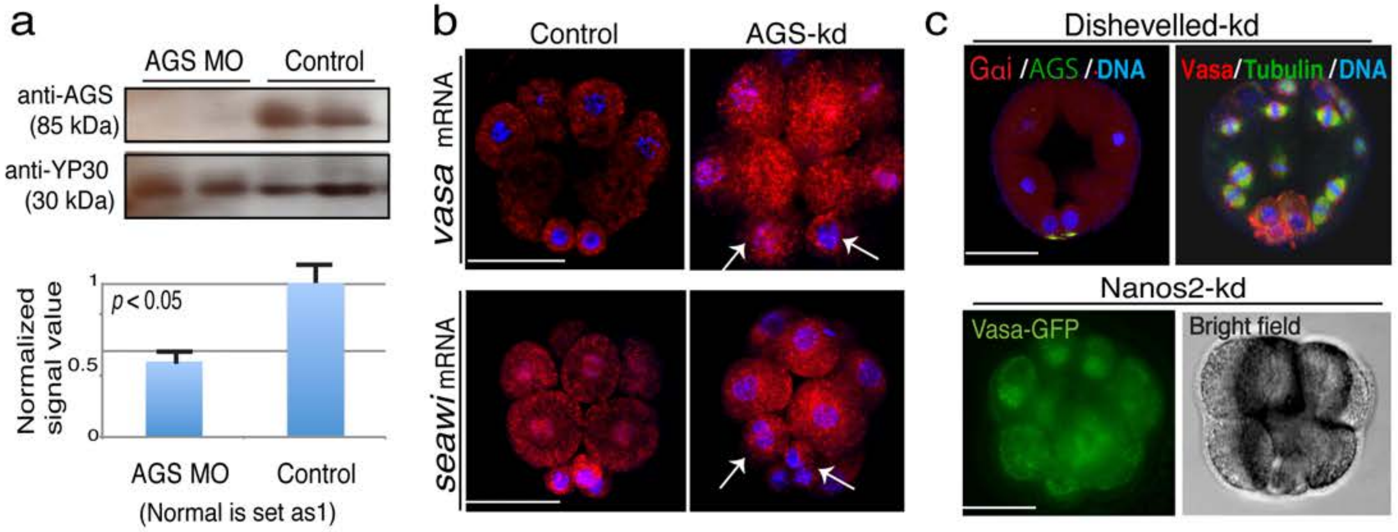

Supplementary Figure 3. AGS-MO kd causes defects in micromere formation and endoderm specification. **a,** Immunoblot analysis of AGS-MO kd. 100 each of 16-cell stage embryos injected with 2mM stock AGS-MO or Control (2mM stock Nanos2-MO) was loaded for each lane. The AGS-MO significantly reduced AGS protein expression, and the remaining of which may be the maternal load. The signal intensity of each band was quantitated by *Image J*. YP30 is consistently expressed during embryogenesis and thus was used here as a standard to normalize the signal. () indicates molecular weight of each protein. **b,** AGS-kd (1mM stock AGS-MO) disturbed enrichment of PGC factors (*vasa* and *seawi*, red) in micromeres (arrows). DNA, blue. **c,** Control MOs showed no developmental toxicity. Immunofluorescence or live imaging of embryos injected with 2mM Dishevelled-MO or Nanos2-MO, respectively. These MOs had no effect on micromere formation at 16-cell stage. Further, no defects were observed at the molecular levels, such as in the localization of AGS (green) and Gαi (red), or of Vasa (red) and Tubulin (green) in Dishevelled-MO embryos. Vasa-GFP expression was also unaffected in the Nanos2-MO embryo co-injected with Vasa-GFP mRNA. The representative phenotypes of 85% or larger in each population are shown (n >20). DNA, blue. Scale bars =50µm.

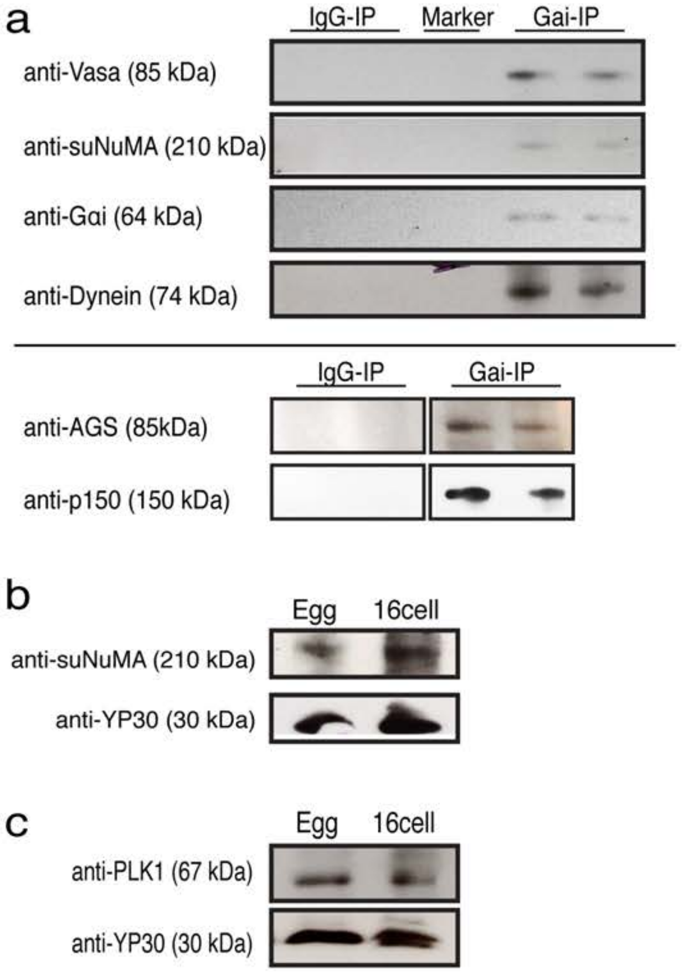

Supplementary Figure 4. Control IP and Immunoblot results. IPs were conducted at least three independent times and two each of the IP-ed samples are shown. Each of the top and bottom groups (demarcated by the line) was processed from a single blot. **a,** IgG-IPed materials showed no specific bands by antibodies used in this paper. **b & c,** Immunoblot analysis of PLK1 (b) and suNuMA (c). 100 each of Eggs or 16-cell stage embryos were loaded for each lane. The expected size of 67 kDa for PLK1 and of 210 kDa for suNuMA was found as a specific band, respectively, suggesting the specificity of each antibody. YP30 is consistently expressed during embryogenesis and thus was used here as a standard to normalize the signal. () indicates molecular weight of each protein.

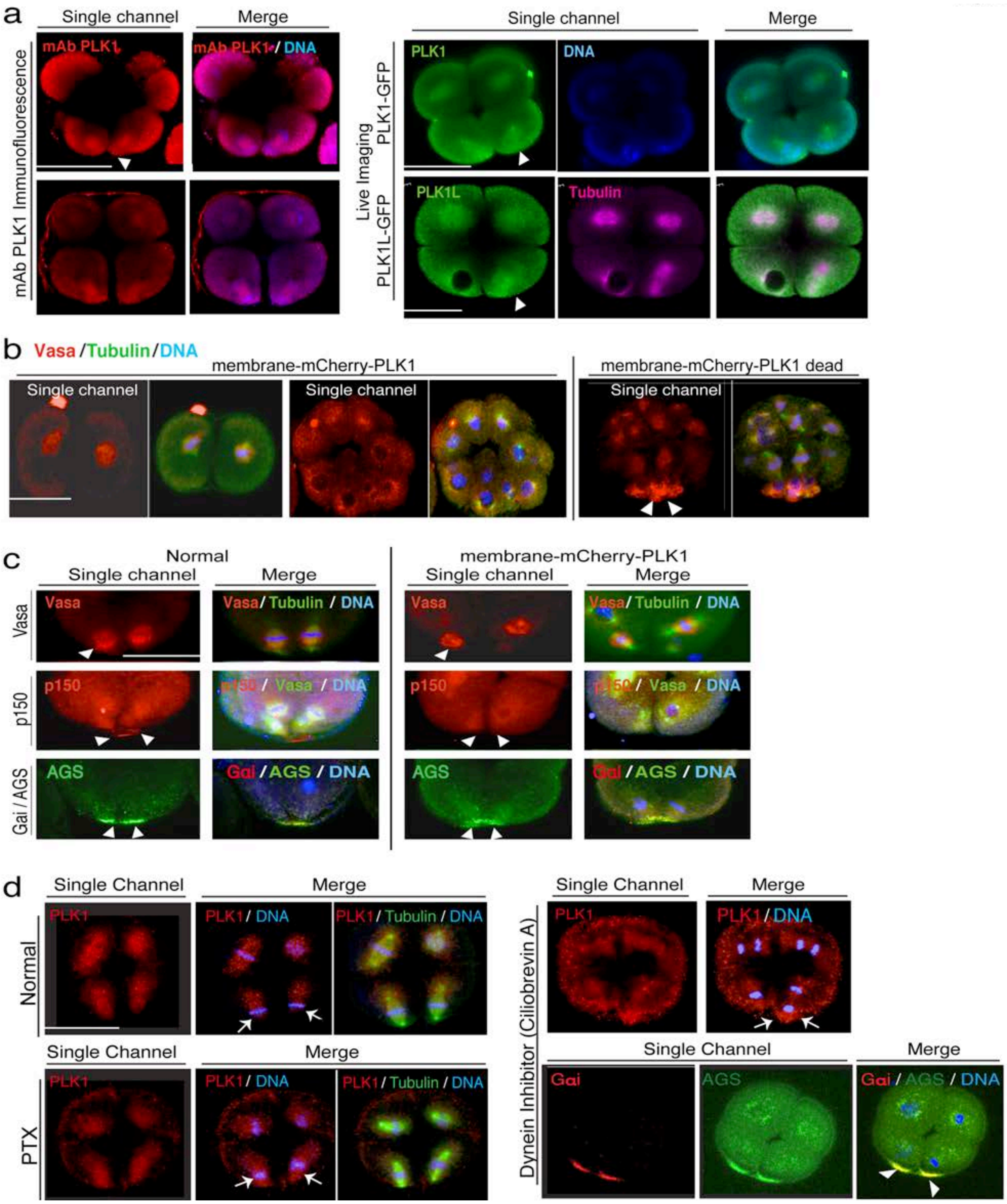

Supplementary Figure 5. Localization and function of PLK1 during embryogenesis. **a,** PLK1 immunofluorescence with another Human PLK1 antibody (mouse monoclonal) in PFA fixation and human PLK1-GFP live imaging (counterstained by Hoechst). Typical PLK1 distributions over the spindle of every blastomere, suggesting human PLK1 mimics the localization of sea urchin PLK1. **b,** membrane-mCherry-PLK1 mRNA or membrane-mCherry-PLK1-Kinase Dead (PLK1-dead) was injected, and immunostained to visualize Vasa (red) and Tubulin (green). Membrane-mCherry-PLK1 randomized cell division orientations and planes and inhibited micromere formation, while membrane-mCherry-PLK1-dead did not. **c,** A vegetal half of the 8-16 cell stage embryos injected with a lower dose (0.25 μg/μl stock) of membrane-mCherry-PLK1 to inhibit micromere formation at the 16-cell stage. In these embryos, proper chromosome segregation was perturbed, centrosome-anchoring to the vegetal cortex was compromised, and Vasa distribution became symmetric at the micromere-side of the spindle (Vasa, arrowhead). Cortical Dynein (stained by anti-Dynactin p150 antibody) signal was also diminished (p150, arrowheads), whereas AGS/Gαi localization at the cortex was unaffected (AGS/Gαi, arrowheads). DNA, blue. **d,** In the PTX (n=41) or Ciliobrevin A-treated (n=25) embryos, at 8-16 cell stage, PLK1 localization was extended over the micromere-side of the spindle that is depleted in the Normal embryos (arrows), whereas Gαi /AGS localization was unaffected (arrowheads). Unless individually indicated, the representative phenotypes of 70% or larger in each population are shown (n > 15) Scale bars = 50μm.

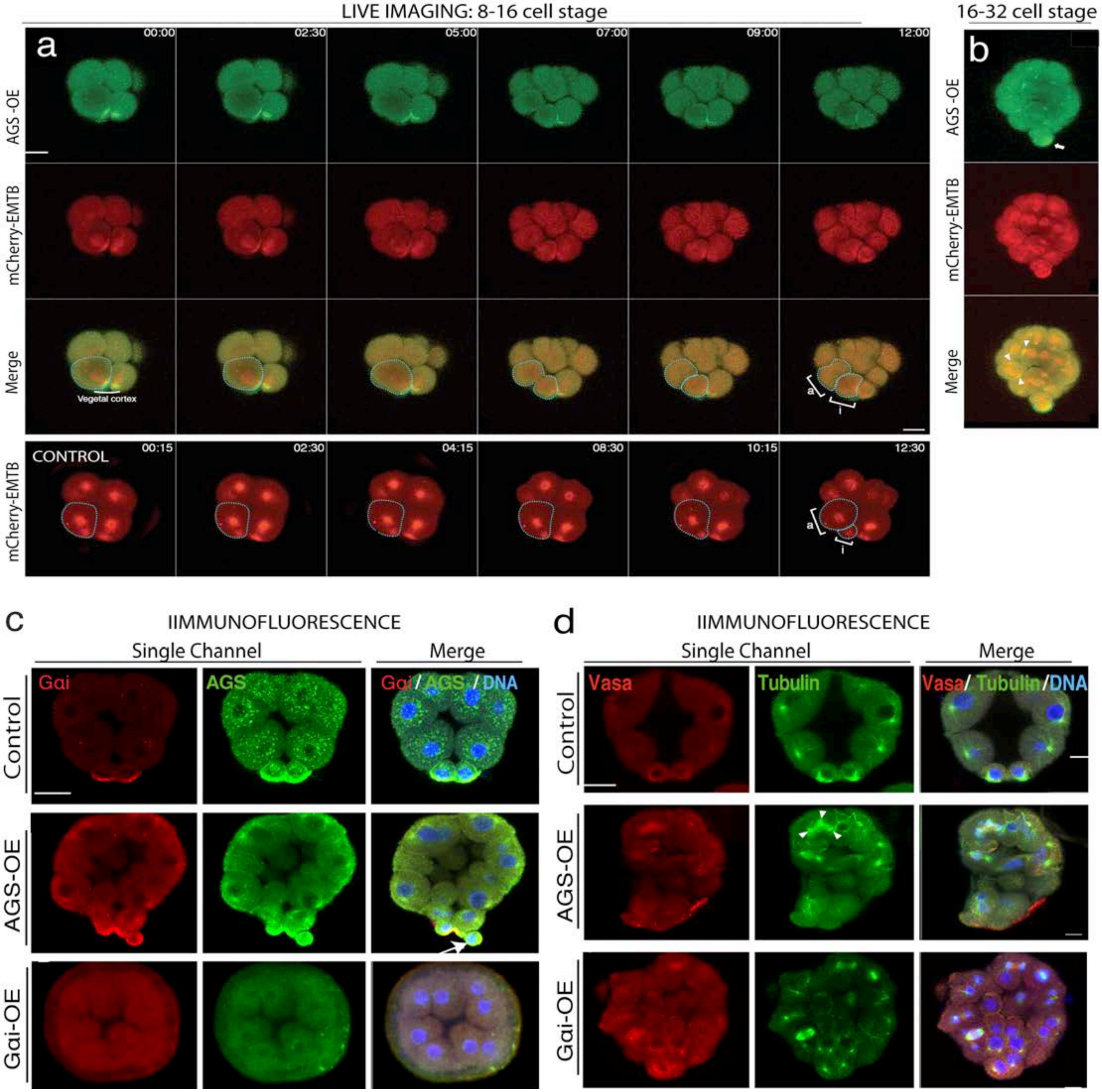

Supplementary Figure 6. AGS co-localizes with microtubules at the cortex in AGS-overexpressing embryos both in live (a, b) and fixed samples (c, d). **a,** In AGS-Overexpressing (0.5μg/μl stock) embryos, AGS-GFP (green) was enriched at the vegetal cortex where the mitotic spindle was anchored during micromere formation. mCherry-EMTB (red) visualizes microtubule dynamics. Division of the vegetal blastomeres resulted in a macromere (a) and a micromere (i) that were similar in size, in which the micromere/macromere ratio was approximately 82%. (Control) Embryos injected only with mCherry-EMTB that underwent normal asymmetric cell division, in which the micromere/macromere diameter ratio was approximately 1:2. Over 70% embryos showed this phenotype, n= 15 **b,** Blastomeres of a 32-cell stage embryo overexpressing AGS (0.5μg/μl stock) lacked attachment, especially blastomeres that were enriched in AGS-GFP (green, arrow). AGS-GFP was enriched at the spindle poles (red, mCherry-EMTB) where they were anchored to the blastomere cortex (arrowheads). Localization at the spindle poles was maintained throughout cell division. **c,** AGS-overexpression (1μg/μl stock) induced poor attachment of AGS and Gαi enriched blastomeres to the rest of the embryo (arrow). Approximately, 50% embryos showed this phenotype, n=20. On the contrary, Gαi-OE inhibited asymmetric cell division of the vegetal blastomeres during micromere formation. Spindles of the vegetal blastomeres were oriented horizontally, rather than vertically, which is the normal spindle orientation during micromere formation. Approximately 80% of embryos showed phenotype, n=21. **d,** AGS-OE embryos (n=16) often showed more than two spindle poles (arrowheads), while Gαi-OE embryos (n=18) showed normal spindle morphology. Approximately, 50% embryos showed this phenotype, n=15. The endogenous AGS, Gαi, and Tubulin signals were detected by anti-AGS, anti-Gαi and anti-Tubulin antibodies, respectively.
Scale bars =20μm.

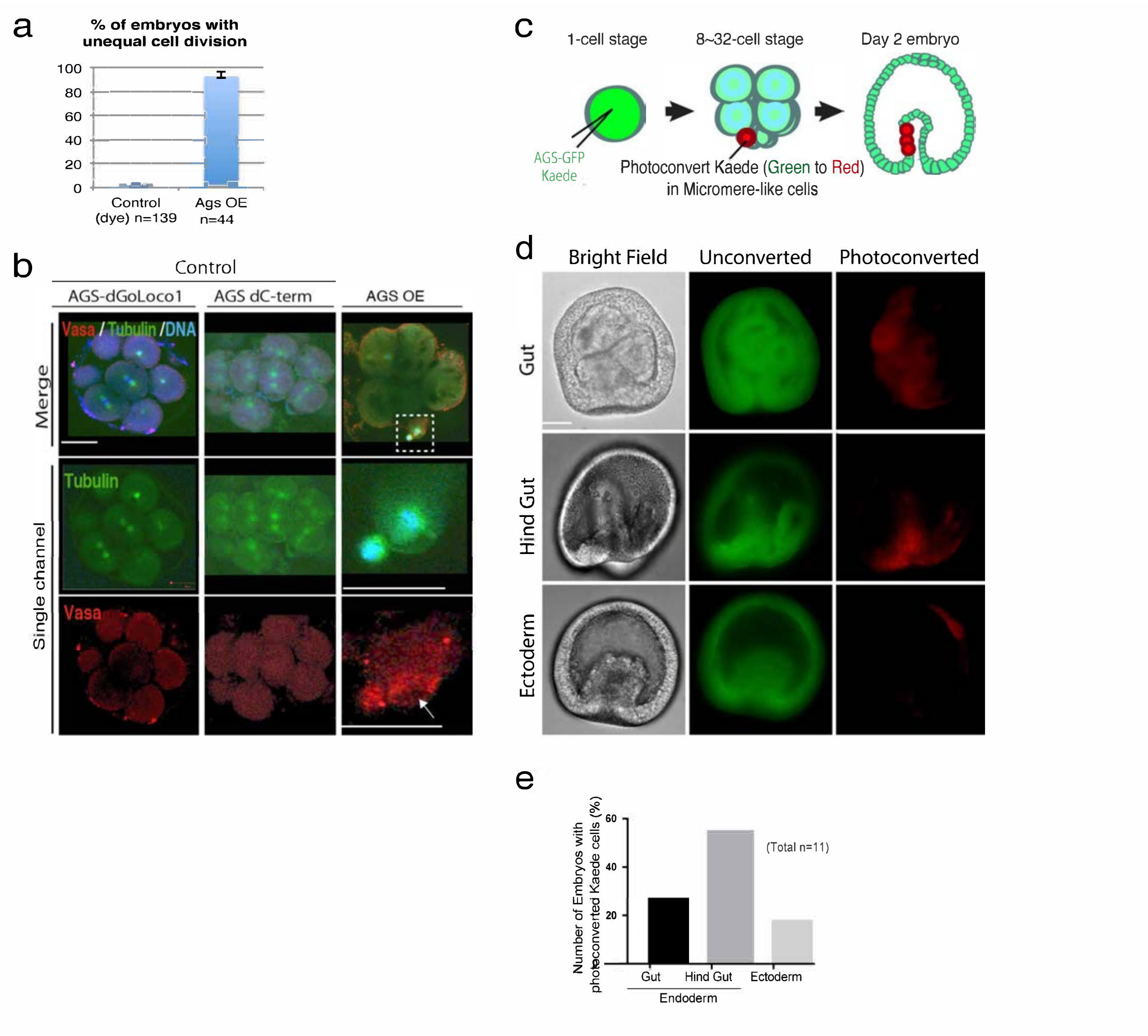

Supplementary Figure 7. Sea urchin AGS (SpAGS) induces asymmetric cell divisions, and the lineage tracing of the micromere-like cells in the sea star embryos. **a,** Sea urchin AGS overexpression (AGS OE) in sea star embryos induced random asymmetric cell divisions from 2 to 16-cell stage, whereas the control embryos injected with dye showed no significantly altered phenotype. n= indicates the total number embryos scored. **b,** Immunofluorescence images of sea star embryos expressing 1.5µg/µl stock of SpAGS-GFP mRNA (OE) or control mRNAs at early embryogenesis. In the experimental group, Vasa (red) was enriched on the spindle of a micromere-like cell undergoing further asymmetric cell division (arrow). A white squared region is enlarged in bottom row image. Approximately, 30% embryos showed this phenotype, n=20. Tubulin, green; DNA, blue. Scale bars = 50µm. **c-e,** 1.5µg/µl stock of SpAGS-GFP mRNA and Kaede-mRNA was injected into unfertilized eggs and the micromere-like cells were photoconverted into red for 0.8 second with 12% UV laser during 8~32 cell stages (c). At Day 2, embryos were scored for each category based on where the photoconverted Kaede (red) signal was found (d-e). At the occasion when the signal was scattered in various tissues, the embryo was counted for each category. Each image indicated the representative phenotype and n indicates the total number of embryos scored in the graph. Scale bars = 50µm.

Table S1. Summary of molecules described in this study

Molecule Name	Background information	Localization patterns in the Sea urchin embryo
Vasa	Vasa is a conserved RNA helicase implicated in translational regulation[1-4]. In the sea urchin, it is perinuclear during S-phase (the sea urchin embryo has no G-phase during early embryogenesis) and is evenly distributed over the spindle complex during M-phase in every blastomere until the 8-cell stage (see Figure 1A)[5]. This localization of Vasa at perinuclear /spindle sites appears to be regulated by Importin, which shuttles various proteins and mRNAs from the cytoplasm to nucleus, but is maintained by microtubules once Vasa is docked to the nuclear envelope[6].	• Vasa is perinuclear during S-phase and on the spindle during M-phase of every blastomere during early embryogenesis.<br><br>• Vasa becomes enriched in the micromeres at 8-16 cell stage and into small micromeres at 16-32 cell stage of each asymmetric cell division.
AGS (or LGN /Pins) and Gαi	AGS (activator of G-protein signaling; orthologs known as Pins in Drosophila and LGN in mammals)[7] contains conserved motifs of a G-protein regulator (GPR) in the C-terminus and of tetratricopeptide repeat (TPR) motifs in the N-terminus. The GPR motifs interact with Gα subunits of heterotrimeric G-proteins[8]. Mammals appear to have two Pins orthologs, called LGN and AGS3, yet only a single AGS has been reported in the sea urchin, which was reported to interact with Gαi by yeast two hybrid screening[9, 10].<br>AGS homologues functioning in asymmetric divisions has so far been detected majorly in specific types of differentiated cells such as *Drosophila* neuroblasts and sensory organ precursor cells and Mouse neuroepithelial and skin progenitors[11]. On the other hand, analysis in human cell culture suggests a more general role of AGS/LGN in cell division via organization of mitotic spindles through interaction with NuMA[12, 13]. In mammals it is also known that LGN alone but not AGS3 is a contributor to asymmetric cell division. This conclusion was based on asymmetric localization of LGN in a cell cycle-dependent manner contrasted to uniform localization of AGS3 in human neural progenitor cells[14]. The function of AGS3 in human cells or the functional contributions of AGS in development of any embryo are poorly understood.	• Both AGS and Gαi localize to the vegetal cortex only during asymmetric cell divisions.<br><br>• AGS is also detectable on the spindle of all blastomeres.

NuMA	NuMA (Nuclear Mitotic Apparatus) is a mammalian high molecular weight (238 kDa) protein, comprised of globular head and tail domains separated by a 1500 amino acid discontinuous coiled-coil[15]. It is known to localize in the nucleus and perinuclear region during Interphase and on the spindle during M-phase[16, 17]. It forms a protein complex with LGN/Gαi at the cortex but also interacts with Dynein-Dynactin motor complex on the astral microtubules. Cortical LGN/Gαi can thus pull the spindle toward the cortex through NuMA-Dynein, regulating spindle positioning and asymmetric cell division[7]. The C-terminus of NuMA contains tubulin-binding and LGN-binding sites and a 100 amino acid stretch that directly binds and bundles microtubules[18, 19].<br>NuMA is not highly conserved in sequence even among vertebrates yet animals in each phylum appears to have a similar scaffold protein, called Mud in *Drosophila* or Lin-5 in *C. elegans*[11].	• NuMA is perinuclear and nuclear during S-phase and on centrosomes and the spindle during the M-phase of every blastomere independent of developmental stage.
PLK1	PLK1 (Polo-like kinase 1) is a serine⁄threonine kinase that is thought to function in cell mitosis by phosphorylating various proteins. The recent report in human cells (Kiyomitsu and Cheeseman, 2012) demonstrated that PLK1 on the spindle-pole regulates Dynein localization by controlling the interaction between Dynein/ Dynactin and its upstream cortical targeting factors NuMA and LGN. When the spindle pole is far from the cell cortex, Dynein-NuMA-LGN complex is stabilized and pulls on astral microtubules to shift the spindle towards the cortex. When the spindle comes close to the cell cortex, on the other hand, NuMA is phosphorylated and Dynein and Dynactin is released from the NuMA-LGN complex at the cortex, releasing the pulling force at the cortex[20].<br>PLK1 is relatively conserved among organisms (Fig. S7A). In other organisms, PLK1 is localized on the spindle complex with enrichment in centrosomes[21-25].	• PLK1 localizes on centrosome and spindle during M-phase of every blastomere independent of developmental stage.<br>• PLK1 is displaced from micromere-side of spindle during asymmetric cell division at 8-16 cell stage.

Supplementary Table 2. SpAGS Blast with Human LGN and AGS3

```
HumanLGN            MREDHSFHVRYRMEASCLELALEGERLCKSGDCRAGVSFFEAAVQVGTEDLKTLSAIYSQ 60
HumanAGS3           ------------MEASCLELALEGERLCKAGDFKTGVAFFEAAVQVGTEDLKTLSAIYSQ 48
SPU_009218.1        ----------MQSEASCMELALEGERLCKAGNCREGVRYLEAAVEVGTDDLKTLSAIYSQ 50
                                 ****:***********:*: : ** ::****:***:***********

HumanLGN            LGNAYFYLHDYAKALEYHHHDLTLARTIGDQLGEAKASGNLGNTLKVLGNFDEAIVCCQR 120
HumanAGS3           LGNAYFYLKEHGRALEYHKHDLLLARTIGDRMGEAKASGNLGNTLKVLGRFDEAAVCCQR 108
SPU_009218.1        LGNAYFYLEDYGKALTYHKHDLTLATSIGDRLGEAKASGNIGNTLKVLGKFDEAICCCQR 110
                    ********.::.:** **:*** ** :***::********:********.****  ****

HumanLGN            HLDISRELNDKVGEARALYNLGNVYHAKGKSFGCPGP---QDVGEFPEEVRDALQAAVDF 177
HumanAGS3           HLSIAQEQGDKVGEARALYNIGNVYHAKGKQLSWNAANATQDPGHLPPDVRETLCKASEF 168
SPU_009218.1        HLDISRELSEKVGEGRALYNLGNVYHAKGKHAGRSGH---QDPGDFPQEVTDCLKQAIQF 167
                    **.*::* .:****.*****:*********  .  .    ** *.:* :* : *  * :*

HumanLGN            YEENLSLVTALGDRAAQGRAFGNLGNTHYLLGNFRDAVIAHEQRLLIAKEFGDKAAERRA 237
HumanAGS3           YERNLSLVKELGDRAAQGRAYGNLGNTHYLLGNFTEATTFHKERLAIAKEFGDKAAERRA 228
SPU_009218.1        YEANLAIVRELGDRAAQGRACGNLGNTHYLLGNFETAIQFHTERLAIAKEFGDKAAERRA 227
                    ** **::*  ********** *************  *   * :** **************

HumanLGN            YSNLGNAYIFLGEFETASEYYKKTLLLARQLKDRAVEAQSCYSLGNTYTLLQDYEKAIDY 297
HumanAGS3           YSNLGNAHVFLGRFDVAAEYYKKTLQLSRQLRDQAVEAQACYSLGNTYTLLQDYERAAEY 288
SPU_009218.1        YSNLGNACVFMVQFEVAAKYYKKSLHIARQLGELAMEAQACYSLGNTYTLLREYEKAVEY 287
                    ******* :*: .*:.*::****:* ::*** : *:***:***********::**:* :*

HumanLGN            HLKHLAIAQELNDRIGEGRACWSLGNAYTALGNHDQAMHFAEKHLEISREVGDKSGELTA 357
HumanAGS3           HLRHLLIAQELADRVGEGRACWSLGNAYVSMGRPAQALTFAKKHLQISQEIGDRHGELTA 348
SPU_009218.1        HSRHMEIAQQLNDRVGEGRACWSLGNAHTSLGNHEKALHYATLHLQISREVGDRTGEVTA 347
                    * :*: ***:* **:************:.::*.  :*: :*  **:**:*:**: **:**

HumanLGN            RLNLSDLQMVLGLSYSTNNSIMSENTEIDSSLNGVRPKLGRRHSMENMELMKLTPEKVQN 417
HumanAGS3           RMNVAQLQLVLGRLTSPAASEKPDLAGYEA--QGARPKRTQRLSAETWDLLRLPLEREQN 406
SPU_009218.1        KMNLQDLQTLFGISTADLSEVSTTVQTPLQESKGARPR--RRRSMENLELVAMTPEKKTE 405
                    ::*: :** ::*   :   .  .         :*.**:  :* * *. :*: :. *:  :

HumanLGN            WNSEILAKQKPLIAKPSAKLLFVNRLKGKKYKTN--SSTKVLQDASNSIDHRIPNSQRKI 475
HumanAGS3           GDSHHSGDWRGPSRDSLPLPVRSRKYQEGPDAER--RPREGSHSPLDSADVRVHVPRTSI 464
SPU_009218.1        IVQVPKRKIRPGSKLKLKNGGESKDKQSHKDSKESVSSAKNSAPAPSKAKSQTSARKGGE 465
                      .    . :             .  :      .   . :    . .. . :    :

HumanLGN            S-ADTIGDEGFFDLLSRFQSNRMDDQRCCLQEKNCHTASTTTSSTPPKMMLKTSSVPVVS 534
HumanAGS3           PRAPSSDEECFFDLLTKFQSSRMDDQRCPLDDGQAGAAEATAAPTLEDRIAQPSMT--AS 522
SPU_009218.1        PIQMLDDADNFFEALSRFQSNRMDEQRCSFGRLQKKLADEEGN---------------GL 510
                    .     . : **: *::***.***:*** :   :   *.

HumanLGN            PNTDEFLDLLASSQSRRLDDQRAS----FSNLPGLRLTQNSQSVLSHLMTNDNKEADEDF 590
HumanAGS3           PQTEEFFDLIASSQSRRLDDQRAS----VGSLPGLRITHSNAGHLRG--HGEPQEPGDDF 576
SPU_009218.1        PEKEELLDEIAKLQGSRLNEQRAFSVKRLPGLPGLRANEDVVGKLLA--KGERAEPDDDF 568
                    *:.:*::* :*. *. **::***     . .***** ...  . *     .:  *..:**

HumanLGN            FDILVKCQGSRLDDQRCAPPPATTKGPTVPDEDFFSLILRSQGKRMDEQRVLLQRDQNRD 650
HumanAGS3           FNMLIKYQSSRIDDQRCPPPDVLPRGPTMPDEDFFSLIQRVQAKRMDEQRVDLAGGPEQ- 635
SPU_009218.1        FEMIIRCQGARIEDQRSTLP-IQAPAPTVPDEDFFSLIQRIQSKRIEEQRSIAPWEKGSG 627
                    *::::: *.:*::***.. *   . .**:********* * *.**::***

HumanLGN            T---DFGLKDFLQNNALLEFKNSGKKSADH- 677
HumanAGS3           ------------GAGGPPEPQQQCQPGAS-- 652
SPU_009218.1        TSCVCFYDYDTSRTEGTACCVYSRPVQESHL 658
```

* The sea urchin AGS (SPU_009218.1) is more similar to the human ortholog AGS3 (gb|AAO17260) than to LGN (gb|AAB40385). Alignment was performed by NCBI blast. The N-terminus is considered responsible for its function and the C-terminus is considered responsible for its cortical localization.

Supplementary Table 3. Alignment of Sp Gai with Human Gai

```
>SPU_013414
Query-Human Gai Subject-SpGai

Query: 1   MGCTLSAEDKAAVERSKMIDRNLREDGEKAAREVKLLLLGAGESGKSTIVKQMKIIHEAG 60
           MGC  SAEDKAA ERSKMIDRNLR +GEKAAREVKLLLLGAGESGKSTIVKQMKIIHE G
Sbjct: 1   MGCATSAEDKAAAERSKMIDRNLRLEGEKAAREVKLLLLGAGESGKSTIVKQMKIIHEEG 60

Query: 61  YSEEECKQYKAVVYSNTIQSIIAIIRAMGRLKIDFGDSARADDARQLFVLAGAAEEGFMT 120
           YSEE+C+QYK VVYSNTIQS+IAIIRAMG LKIDFGD+ RADDARQLF LAG AEEG ++
Sbjct: 61  YSEEDCRQYKPVVYSNTIQSMIAIIRAMGSLKIDFGDTERADDARQLFALAGQAEEGELS 120

Query: 121 AELAGVIKRLWKDSGVQACFNRSREYQLNDSAAYYLNDLDRIAQPNYIPTQQDVLRTRVK 180
            ELA V+KRLW DSGVQACF+RSREYQLNDSA+YYLN LDR++ P YIPTQQDVLRTRVK
Sbjct: 121 TELAAVMKRLWADSGVQACFSRSREYQLNDSASYYLNALDRLSAPGYIPTQQDVLRTRVK 180

Query: 181 TTGIVETHFTFKDLHFKMFDVGGQRSERKKWIHCFEGVTAIIFCVALSDYDLVLAEDEEM 240
           TTGIVETHFTFK+LHFKMFDVGGQRSERKKWIHCFEGVTAIIFCVALS YDLVLAEDEEM
Sbjct: 181 TTGIVETHFTFKELHFKMFDVGGQRSERKKWIHCFEGVTAIIFCVALSAYDLVLAEDEEM 240

Query: 241 NRMHESMKLFDSICNNKWFTDTSIILFLNKKDLFEEKIKKSPLTICYPEYAGSNTYEEAA 300
           NRMHESMKLFDSICNNKWFT+TSIILFLNKKDLFEEKI+KSPLTIC+PEY GSNTYEEAA
Sbjct: 241 NRMHESMKLFDSICNNKWFTETSIILFLNKKDLFEEKIQKSPLTICFPEYTGSNTYEEAA 300

Query: 301 AYIQCQFEDLNKRKDTKEIYTHFTCATDTKNVQFVFDAVTDVIIKNNLKDCGLF 354
           AYIQ QFEDLNKRKD KEIYTHFTCATDT N+QFVFDAVTDVIIKNNLKDCGLF
Sbjct: 301 AYIQMQFEDLNKRKDQKEIYTHFTCATDTNNIQFVFDAVTDVIIKNNLKDCGLF 354
```

Supplementary Table 4. SpPLK1 alignment with Human PLK1

```
SpPLK1         --------------------------MASRHKEDLRVKEVPDVVTDPSTGKTYTKGRFLG
HumanPLK1      MSAAVTAGKLARAPADPGKAGVPGVAAPGAPAAAPPAKEIPEVLVDPRSRRRYVRGRFLG
                                           .       .**:*:*:.** : : *.:*****

SpPLK1         KGGFAKCYELTDDATKQIFAGKVVSKALLVKPHQKDKMTMEIHIHKSLHHRHVVGFHSFF
HumanPLK1      KGGFAKCFEISDADTKEVFAGKIVPKSLLLKPHQREKMSMEISIHRSLAHQHVVGFHGFF
               *******:*::*  **::****:* *:**:****::**:*** **:** *:******.**

SpPLK1         EDKENVYVLLELCRRRSLMELHKRRKAITEPETRYFMRQCILACQYLSKTKVIHRDLKLG
HumanPLK1      EDNDFVFVVLELCRRRSLLELHKRRKALTEPEARYYLRQIVLGCQYLHRNRVIHRDLKLG
               **:: *:*:*********:********:****:**::** :*.**** :.:*********

SpPLK1         NLFIDDNMELKVGDFGLATKVDFSGERKKTLCGTPNYIAPEVLSKKGHSYEVDLWSLGCI
HumanPLK1      NLFLNEDLEVKIGDFGLATKVEYDGERKKTLCGTPNYIAPEVLSKKGHSFEVDVWSIGCI
               ***:::::*:*:*********::.*************************:***:**:***

SpPLK1         MYTLLVGKPPFETQSLKDTYQRIKRNEYRVPSHVSTPARNLIVKLLKNDPTQRPHIDILL
HumanPLK1      MYTLLVGKPPFETSCLKETYLRIKKNEYSIPKHINPVAASLIQKMLQTDPTARPTINELL
               *************..**:** ***:*** :*.*:.  * .** *:*:.*** ** *: **

SpPLK1         QDEFFTTGYLPPQLPTTCLTMAPRFQVPVSS----GRRPLLEVNGQDENVPPGREQS-GK
HumanPLK1      NDEFFTSGYIPARLPITCLTIPPRFSIAPSSLDPSNRKPLTVLNKGLENPLPERPREKEE
               :*****:**:* :** ****: ***.:  **    .*:**  :*   **  * * :.  :

SpPLK1         PHRKHSDRKENGGGKPKVYQPKEDHLASLKVHLTSVVAAKPSEKADIRLDEAEDPAAAPI
HumanPLK1      PVV-----RE-------TGEVVDCHLSDMLQQLHSVNASKPSERGLVRQEEAEDPACIPI
               *       :*       . :  : **:.:  :* ** *:****:. :* :******. **

SpPLK1         LWVSKWVDYSDKYGLGYQLCDGSVGVLFNDSTRLLLHANADTLEYIERDGNEKYCRLGSY
HumanPLK1      FWVSKWVDYSDKYGLGYQLCDNSVGVLFNDSTRLILYNDGDSLQYIERDGTESYLTVSSH
               :********************.************:*: :.*:*:******.*.*  :.*:

SpPLK1         DSTLHKKVTLLKYFRNYMSEHLLKAGAAMTPRESDSMARLPFLQSWFRTKSAIVLHLSNG
HumanPLK1      PNSLMKKITLLKYFRNYMSEHLLKAGANITPREGDELARLPYLRTWFRTRSAIILHLSNG
                .:* **:******************* :****.*.:****:*::****:***:******

SpPLK1         TVQINFFEDHTKLIVCPMMGAATYIDAKRNFRTFRLNLIEKHGCTPDLYDRIKYANNMVK
HumanPLK1      SVQINFFQDHTKLILCPLMAAVTYIDEKRDFRTYRLSLLEEYGCCKELASRLRYARTMVD
               :******:******:**:*.*.**** **:***:**.*:*::**  :* .*::**..**.

SpPLK1         NMLDKKTTTTTAAAVPAH
HumanPLK1      KLLSSRSASNRLKAS*--
               ::*..::::.   *
```

*Yellow-highlighted region: Antigen region of Rabbit polyclonal Antibody to PLK1
**Underlined region: Antigen region for Mouse monoclonal Antibody to PLK1
Orange sequences are lacking in SpPLK1L

Supplementary Table 5. Sea urchin SpAGS alignment with

Sea star PmAGS `Query=Urchin; Sbjt=Pm`

```
Query: 4   EASCMELALEGERLCKAGNCREGVRYLEAAVEVGTDDLKTLSAIYSQLGNAYFYLEDYGK 63
           +ASCMELALEGERLCKAGNC+ GV+Y EAAVEVGTDDL+TLSAIYSQLGNAYFYL+DY +
Sbjct: 18  DASCMELALEGERLCKAGNCKSGVQYFEAAVEVGTDDLRTLSAIYSQLGNAYFYLQDYTR 77

Query: 64  ALTYHKHDLTLATSIGDRLGEAKASGNIGNTLKVLGKFDEAICCCQRHLDISRELSEKVG 123
           AL YH+HDLTLA ++GD +GEAKASGN+GNTLKVLGKFDEAI CCQRHLDISREL ++VG
Sbjct: 78  ALEYHRHDLTLACTLGDHIGEAKASGNLGNTLKVLGKFDEAIVCCQRHLDISRELGDRVG 137

Query: 124 EGRALYNLGNVYHAKGKHAGRSGHQDPGDFPQEVTDCLKQAIQFYEANLAIVRELGDRAA 183
           EGRALYNLGNVYHAKGKHAGR+GHQDPGDFP+EV  CL++A++FYE NLAIV+ L D+AA
Sbjct: 138 EGRALYNLGNVYHAKGKHAGRAGHQDPGDFPEEVIGCLRKAVEFYEMNLAIVKSLVDKAA 197

Query: 184 QGRACGNLGNTHYLLGNFETAIQFHTERLAIAKEFGDKAAERRAYSNLGNACVFMVQFEV 243
           QGRACGNLGNTHYLLGNFETAI FH ERL+IAKEFGDKAAERRAYSNLGNACVFM  FE
Sbjct: 198 QGRACGNLGNTHYLLGNFETAIGFHKERLSIAKEFGDKAAERRAYSNLGNACVFMGDFET 257

Query: 244 AAKYYKKSLHIARQLGELAMEAQACYSLGNTYTLLREYEKAVEYHSRHMEIAQQLNDRVG 303
           AA YYKK+LHIAR+LG++A+EAQACYSLGNT+TLLR+YE AV+YH RH  IAQ+L DRVG
Sbjct: 258 AADYYKKTLHIARRLGDIAIEAQACYSLGNTFTLLRDYETAVDYHERHHRIAQKLKDRVG 317

Query: 304 EGRACWSLGNAHTSLGNHEKALHYATLHLQISREVGDRTGEVTAKMNLQDLQTLFGISTA 363
           EGRACWSLGNAHT+L NHEKALHYATLHLQISREVGD+TGE+TA+MNL DL+T+ G+
Sbjct: 318 EGRACWSLGNAHTALQNHEKALHYATLHLQISREVGDKTGELTAQMNLADLRTVLGLDKD 377

Query: 364 DLSE---VSTTVQTPLQESKGARPRRRRSMENLELVAMTPEKKTEIVQV-PKRKIRPGSK 419
             S+   +    +  L+E K    +RR+SME LELV MTP+K         K K   G+K
Sbjct: 378 PCSQDGLLQNASKKTLKE-KELNRQRRQSMERLELVTMTPDKNAAAATANGKPKQSAGAK 436

Query: 420 LKLKNGGESKDKQSHKDSKEXXXXXXXXXXXXXXXXXXXXXRKGGE---PIQMLDDADNF 476
            K K  G +K+K S K S                       +   +     + +DD D+F
Sbjct: 437 FKRK--GSNKEKLSRKSSNTSSTSSSSGESAACLRTENVNIQVSPQQRSSPEFMDDDDSF 494

Query: 477 FEALSRFQSNRMDEQRCSFGRLQKKLAD---EEGNGLPEKEELLDEIAKLQGSRLNEQRA 533
           F+ LSRFQ  RMDEQRCSF R+Q K  +   +E    P KEEL+++IA  Q SRLNEQR+
Sbjct: 495 FDVLSRFQGKRMDEQRCSFNRMQDKQRERENDEDRDYPMKEELMNQIASFQRSRLNEQRS 554

Query: 534 FSVKRLPGLPGLRANEDVVGKLLAKGERAEPDDDFFEMIIRCQGARIEDQRSTLPIQAPA 593
            S+  LPGL     NE+V+G+LL KG +  PDDDFF+M++RCQG+RI DQRS  P+  PA
Sbjct: 555 -SICNLPGLK--TNNEEVLGQLLQKGNQGVPDDDFFDMLMRCQGSRINDQRSEPPVLQPA 611

Query: 594 PTVPDEDFFSLIQRIQSKRIEE
618
           PTVPDEDFF+LIQR+QSKR++ QRS
Sbjct: 612 PTVPDEDFFALIQRVQSKRMDAQRS 636
```

* Sea urchin AGS (SpAGS) and sea star AGS (PmAGS) are similar in the N-terminus but highly variable in the C-terminus in which the putative four GoLoco motifs responsible for its interaction with Gαi at the cortex are present.

Supplementary Table 6. Alignment of echinoderm AGS proteins

A

```
OjAGS               ------------MRANMDMPCMELALEGERLCKMGCCREGVEYFEAAVKVGTDDLKTLSA
SbAGS               ----------------MEASCMELALEGERLCKTGNCREAVRYLEAAVRVGTDDLQTLSA
PpAGS               MRLELEEGRASTNAFKMDASCMELALEGERLCKAGNCKSGVQYFEAAVEVGTDDLRTLSA
PmAGS               MRLELEEGRASTNAFKMDASCMELALEGERLCKAGNCKSGVQYFEAAVEVGTDDLRTLSA
EtAGS(pencil        --------------MQSEASCMELALEGERLCKVGNCREGVRYLEAAVEVGTDDLKTLSA
EpAGS               --------------MQSETSCMELALEGERLCKVGKCSEGVRYLEAAVEEGTDDLKMLSA
SpAGS               --------------MQSEASCMELALEGERLCKAGNCREGVRYLEAAVEVGTDDLKTLSA
SgAGS               --------------MQSEASCMELALEGERLCKAGNCREGVRYLEAAVEVGTDDLKTLSA
LvAGS(sea           --------------MQSEASCMELALEGERLCKAGNCREGVRYLEAAVEVGTDDLKTLSA
                                     :  ************* * * ..*.*:****. *****: ***

OjAGS               IYSQLGNAYFYLQEYGKALEYHRHDLTLASTIGDRLGEAKARGNLGNTLKVLGKFDEAVM
SbAGS               IYSQLGNAYFYLQDYLKALTYHKHDLTLARSIEDRLGEAKASGNIGNTLKVLGKYDEAIL
PpAGS               IYSQLGNAYFYLQDYTRALEYHRHDLTLACTLGDHIGEAKASGNLGNTLKVLGKFDEAIV
PmAGS               IYSQLGNAYFYLQDYTRALEYHRHDLTLACTLGDHIGEAKASGNLGNTLKVLGKFDEAIV
EtAGS(pencil        IYSQLGNAYFYLQDYRKALTYHKHDLTLASSIGDRLGEAKASGNIGNTLKVLGKFDEAIF
EpAGS               IYSQLGNAYFYLQDYVKALTYHKHDLTLATSIGDRLGEAKASGNIGNTLKVLGKFDEAIC
SpAGS               IYSQLGNAYFYLEDYGKALTYHKHDLTLATSIGDRLGEAKASGNIGNTLKVLGKFDEAIC
SgAGS               IYSQLGNAYFYLEDYGKALTYHKHDLTLATSIGDRLGEAKASGNIGNTLKVLGKFDEAIC
LvAGS(sea           IYSQLGNAYFFLEDYGKALTYHKHDLTLATSIGDRLGEAKASGNIGNTLKVLGKFDEAIC
                    **********:*::* :** **:****** :: *::***** **:*********:***:

OjAGS               CCQGHLDISRELGDKMGEARALYNLGNVFHAKGKHSNGSGHQDPGDFPRDVKQSLLTAID
SbAGS               CCQKHLEISKELKDTVGEARALYNLGNVYHAKGKSAGKAGHQEPGDFPETVTSCLKQAIV
PpAGS               CCQRHLDISRELCDRVGEGRALYNLGNVYHAKGKHAGRAGHQDPGDFPEEVIGCLRKAVE
PmAGS               CCQRHLDISRELGDRVGEGRALYNLGNVYHAKGKHAGRAGHQDPGDFPEEVIGCLRKAVE
EtAGS(pencil        CCQRHLDISRELSDKVGEGRALYNLGNVYHAKGKHAGRSGHQDPGDFPEEVTDCLKKAIE
EpAGS               CCERHLVISRELSDKIGEARSLYNLGNVYHAKGKHAGRSGHQDPGDFPQEVTECLKQAIQ
SpAGS               CCQRHLDISRELSEKVGEGRALYNLGNVYHAKGKHAGRSGHQDPGDFPQEVTDCLKQAIQ
SgAGS               CCQRHLDISRELSEKVGEGRALYNLGNVYHAKGKHAGRSGHQDPGDFPQEVTDCLKQAIQ
LvAGS(sea           CCQRHLDISRELSEKVGEGRALYNLGNVYHAKGKHAGRSGHQDPGDFPQEVTDCLKQAIQ
                    **: ** **:** : :**.*:*******:***** :. :***:*****. *  .*  *:

OjAGS               YYEANLKLVRELGDRAAQGRACGNLGNTYYLLGDFDVAIKYHEERLFIAKEFGDKAAERR
SbAGS               YYETNLQIVRELNDRAAQGRACGNLGNTHYLLGNFDLAIKYHEERLSIAKEFGDKPAERR
PpAGS               FYEMNLAIVKSLVDKAAQGRACGNLGNTHYLLGNFDTAIGFHKERLSIAKEFGDKAAERR
PmAGS               FYEMNLAIVKSLVDKAAQGRACGNLGNTHYLLGNFETAIGFHKERLSIAKEFGDKAAERR
EtAGS(pencil        YYQANLAIVRELGDRAAQGRACGNLGNTHYLLGNFDTAIQFHKERLAIAKEFGDKAAERR
EpAGS               FYEANLGIVRELGDRAAQGRACGNLGNTHYLLGNFETAIQYHTERLAIAKEFGDKAAERR
SpAGS               FYEANLAIVRELGDRAAQGRACGNLGNTHYLLGNFETAIQFHTERLAIAKEFGDKAAERR
SgAGS               FYEANLAIVRELGDRAAQGRACGNLGNTHYLLGNFETAIQFHTERLAIAKEFGDKAAERR
LvAGS(sea           FYEANLAIVRELGDRAAQGRACGNLGNTHYLLGNFETAIQFHTERLAIAKEFGDKAAERR
                    :*: ** :*:.* *:*************:****:*: ** :* *** ******** ****

OjAGS               AYSNLGNAHVFKGEFETAADYYKRTLLIARGLADQAIEAQACYSLGNTFTLLRDFEQAVE
SbAGS               AYSNLGNSHVFLGDFGAAAEYYKKTLDVARQLRDVAMEAQACYSLGNTYTLMREYEAAIE
PpAGS               AYSNLGNACVFMGDFETAADYYKKTLHIARRLGDIAIEAQACYSLGNTFTLLRDYETAVD
PmAGS               AYSNLGNACVFMGDFETAADYYKKTLHIARRLGDIAIEAQACYSLGNTFTLLRDYETAVD
EtAGS(pencil        AYSNLGNACVFLVKFESAAEYYKKALHIARQLGESAMEAQACYSLGNTYTLLREYEKAVE
EpAGS               AYSNLGNACVFMVEFDMAAEYYKKALHIARQLAELAMEAQACYSLGNTYTLLREYEKAVE
SpAGS               AYSNLGNACVFMVQFEVAAKYYKKSLHIARQLGELAMEAQACYSLGNTYTLLREYEKAVE
SgAGS               AYSNLGNACVFMVQFEVAAKYYKKALHIARQLGELAMEAQACYSLGNTYTLLREYEKAVE
LvAGS(sea           AYSNLGNACVFMVQFEVAAKYYKKALHIARQLGELAMEAQACYSLGNTYTLLREYEKAVE
                    *******: **  .*  **.***::* :** * : *:***********:**:*::* *::

OjAGS               YHMRHLHIAQHVNDRVGEGRAYWSLGNAYTALKKYEKAIQYASFHLEISKEVGDKTGEII
SbAGS               YHVKHLKIAQQLQDRVGEGRACWSLGNAHAALAHHEKALHYATLHFQISREVGDRTGEVT
PpAGS               YHERHHRIAQKLKDRVGEGRACWSLGNAHTALQNHEKALHYATLHLQISREVGDKTGELT
PmAGS               YHERHHRIAQKLKDRVGEGRACWSLGNAHTALQNHEKALHYATLHLQISREVGDKTGELT
EtAGS(pencil        YHMRHMEIAQQLQDRVGEGRACWSLGNAHTSLGNHEKALHYATLHLQISREVGDRTGEVT
EpAGS               YHMRHMEIAQQLNDRVGEGRACWSLGNAHTSLGSHEKALQYATLHLQISREVGDRTGEVT
SpAGS               YHSRHMEIAQQLNDRVGEGRACWSLGNAHTSLGNHEKALHYATLHLQISREVGDRTGEVT
SgAGS               YHGRHMEIAQELNDRVGEGRACWSLGNAHTSLGNHEKALHYATLHLQISREVGDRTGEVT
LvAGS(sea           YHGRHMEIAQELNDRVGEGRACWSLGNAHTSLGNHEKALHYATLHLQISREVGDRTGEVT
                    ** :* .***.::******** ******:::*  :***::**::*::**:****:***:

OjAGS               AQMNLSDLTRLVGNGFRNEERNGSSICS------REDDGKPRRRSMENLELVRMTPEKNS
SbAGS               AQMNLVDLQTVLGLSKKGPEEE-----------KQGRDARSRRKSMENLEFMSLTPEKNG
PpAGS               AQMNLADLRTVLGLDKDPCSQDGLLQNASKKTLKEKELNRQRRQSMERLELVTMTPDKNA
PmAGS               AQMNLADLRTVLGLDKDPCSQDGLLQNASKKTLKEKELNRQRRQSMERLELVTMTPDKNA
EtAGS(pencil        AQMNLADLKTLFGIGTGDLSHDVTAAC-PPAMQPTKGATRPRRRSMENLELVAMTPEKNT
EpAGS               AQMNLSDLQKIFGLSPGDISNATT----QVPDKATPGARPRRRSSMENLELVAMTPE---
SpAGS               AKMNLQDLQTLFGISTADLSEVSTTV--QTPLQESKGARPRRRRSMENLELVAMTPEKKT
```

```
SgAGS               AKMNLQDLQSLFGISTSDLSDVSTTV--QTPLQETKGARPRRRRSMENLELVAMTPEKKA
LvAGS(sea           AKMNLQDLQSLFGISTSDISDVSTTV--QTPLQETKGARPRRRRSMENLELVAMTPEKKA
                    *:*** **  :.* .    .                     ** ***.**:: :**:

OjAGS               SEKPNKDQVDGKPKGARVKE---KSSKEKLVHAGSNNSIGANKATDPGQVTV------NL
SbAGS               ATAIQPVAKPLKPV---NKNKKKTSSKEKLSRKGSDTSSDSGGSAGSNK---AESN----
PpAGS               AAATANGK-PKQSTGAKFKRK--GSNKEKLSRKSSNTSSTSSSSGDSAACVRTENVNIQV
PmAGS               AAATANGK-PKQSAGAKFKRK--GSNKEKLSRKSSNTSSTSSSSGESAACLRTENVNIQV
EtAGS(pencil        DSA-VPNG-KLKPTGSKSKRRNGKE---KLSRKGSNTSTGSSNSSSSAPS----QASTQA
EpAGS(sand)         ------------------------------------------------------------
SpAGS               EIVQVPKR-KIRP-GSKLKLKNGGESKDKQSHKDSKESVSSAKNSAPAPS----KAKSQT
SgAGS               EINQVPKR-KIRP-GSKIKLKNGGESKDKQSLKHSKEPVSSAKNSAPAPS----KAKTQT
LvAGS(sea           EINQVPKR-KIRP-GSKIKLKNGGESKDKQSHKHSKEPVSSAKNSAPAPS----KAKTRT


OjAGS               ---KPPRKYSSPDFDDSFFDLLSRFQGNRMDEQRMSLPKSNSL-----DKCDTSDEGKEE
SbAGS               -GVKLNGSQLDEDDQNDFFDALSRFQEKRINDQRVSFGLDD-----------TDAANKED
PpAGS               SP-QQRSSPEFMDDDDSFFDVLSRFQGKRMDEQRCSFNRMQDKQRERENDEDRDYPMKEE
PmAGS               SP-QQRSSPEFMDDDDSFFDVLSRFQGKRMDEQRCSFNRMQDKQRERENDEDRDYPMKEE
EtAGS(pencil        SSQKGGEPQEMLDDADNFFEALSRFQSNRMDEQRCSFGRMQRKLQE--DEEANGLPEKEE
EpAGS               ---KGGEPTQMLDDADNFFEALSRFQSNRMDEQRCSFGRLTKKLD---DDEENGLPEKEL
SpAGS               SARKGGEPIQMLDDADNFFEALSRFQSNRMDEQRCSFGRLQKKLA---DEEGNGLPEKEE
SgAGS               SARKGGEPTQMLDDADNFFEALSRFQSNRMDEQRCSFGRLQKKLA---DEEGNGLPEKEE
LvAGS(sea           SARKGGEPTQMLDDADNFFEALSRFQSNRMDEQRCSFGRLQKKLA---DEEGNGLPEKEE
                       :        *  :.**: ***** :*:::** *:                .   **

OjAGS               LMNSIAKFQGKRMNEQRMHV----ASLPGLRGKDEELLSKLK--EASSVPDDDFFDMLIR
SbAGS               LLDEIAKVQGNRLNEQRANV----ELLPGLR-GHREVVDRLLASGDGAVPDDDFFEMLMR
PpAGS               LMNQIASFQRSRLNEQRSSI----CNLPGLKTNNEEVLGQLLQKGNQGVPDDDFFDMLMR
PmAGS               LMNQIASFQRSRLNEQRSSI----CNLPGLKTNNEEVLGQLLQKGNQGVPDDDFFDMLMR
EtAGS(pencil        LLDEIAKLQGSRLNEQRAFSAQRLPGLPGLR-ANENVLGKLLAKGDLAVPDDDFFEMLMR
EpAGS               LLDEIAKLQGSRLNEQRVSVAERLPGLPGLR-ANDKVLGTLLAKGERAVPDDDFFEMLMR
SpAGS               LLDEIAKLQGSRLNEQRAFSVKRLPGLPGLR-ANEDVVGKLLAKGERAEPDDDFFEMIIR
SgAGS               LLDEIAKLQGSRLNEQRASSAQRLPGLPGLR-ANEDVVGKLLAKGDRVEPDDDFFEMIIR
LvAGS(sea           LLDEIAKLQGSRLNEQRASSAQRLPGLPGLR-ANEDVVGKLLAKGDRAEPDDDFFEMIIR
                    *::.**..* .*:****         ****:  . .::. *        ******:*::*

OjAGS               CQGTRIEDQRTDAPERVRAPTVPDEDFFSLISRVQSQRLEEQRCDLPESLSRDMITDEK*
SbAGS               CQGARMEDQRSSLPSSRPAPTVPDEDFFGLIQRIQSRRIEEQRTDAPWERPTKTRES*--
PpAGS               CQGSRINDQRSEPPVLQPAPTVPDEDFFALIQRVQSKRMDAQRSDKGPQDQTK*------
PmAGS               CQGSRINDQRSEPPVLQPAPTVPDEDFFALIQRVQSKRMDAQRSDKGPQEQTK*------
EtAGS(pencil        CQGARIEDQRSTLPMPTPAPTVPDEDFFTLIQRIQSRRIEEQRTIPPWEKGPKPSDK*--
EpAGS               CQGARIEDQRSSLPVAPPAPTVPDEDFFSLIQRIQSKRIEEQRMIAPWEKD--ASCMCFY
SpAGS               CQGARIEDQRSTLPIQAPAPTVPDEDFFSLIQRIQSKRIEEQRSIAPWEKGSGTSCVCFY
SgAGS               CQGARIEDQRSTLPIQVPAPTVPDEDFFSLIQRIQSKRIEEQRTIPPWEKGSGTSCVCFY
LvAGS(sea           CQGARIEDQRSTLPIQVPAPTVPDEDFFSLIQRIQSKRIEEQRTIPPWEKGSGTSCVCFY
                    ***:*::***:  *    ********** **.*:**:*:: **     .

OjAGS               -------------------------
SbAGS               -------------------------
PpAGS               -------------------------
PmAGS               -------------------------
EtAGS(pencil        -------------------------
EpAGS               SYESSTIHGSACCVYRRPIQQTSI*
SpAGS               DYDTSRTEGTACCVYSRPVQESHL*
SgAGS               DYDTSRTEGTACCVYIRPVQESHL
LvAGS(sea           DYDTSHAEGTACCVYIRPVQESHL*
```

B

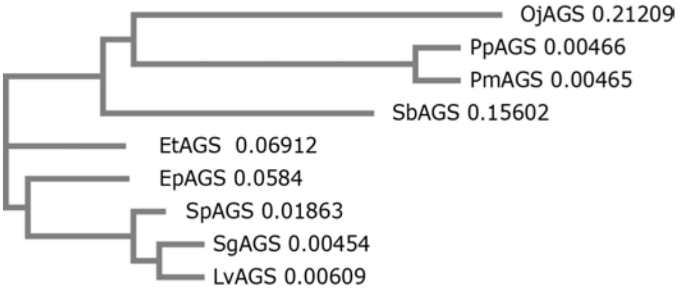

* The protein sequence alignment was analyzed by Clustal Omega. Oj, feather star; Sb, sea cucumber; Pp & Pm, sea star; Et, pencil urchin; Ep, Sand dollar; Sp & Sg & Lv, sea urchin. AGS protein phylogenetic tree (via neighbor joining) was constructed using the sequences presented in A by Clustal Omega. A pairwise identity scores matrix is indicated to the right of each species name.

Supplementary Table 7. Motif prediction of echinoderm AGS proteins

A. The NCBI motif search result of each echinoderm AGS sequence

Sand Dollar

Sand Dollar

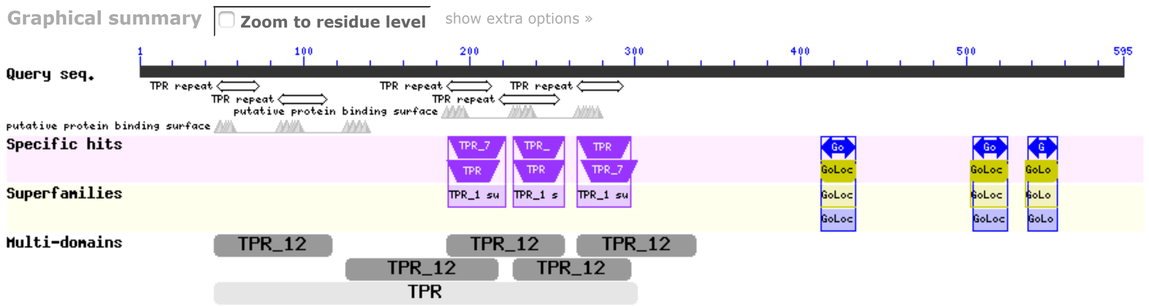

Sea urchin

LvAGS(sea urchin)

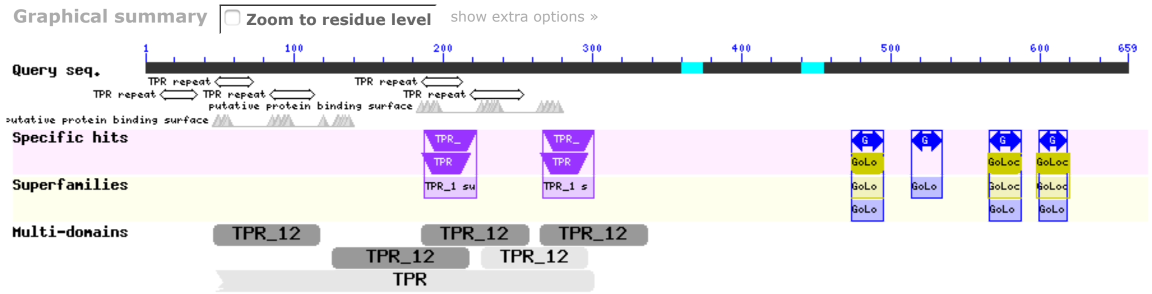

SgAGS (sea urchin)

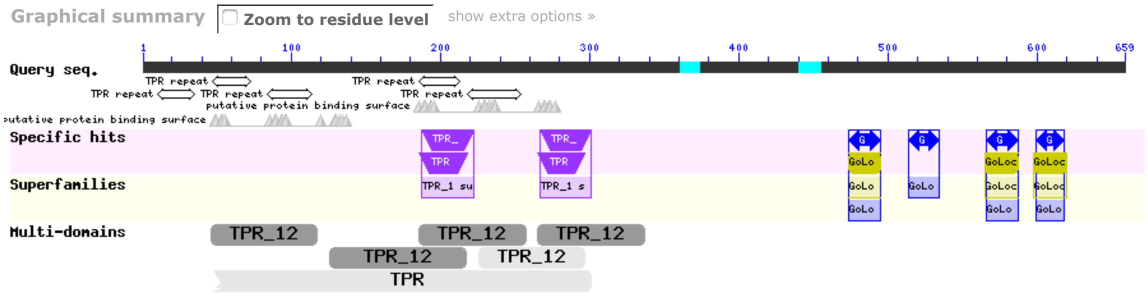

SpAGS

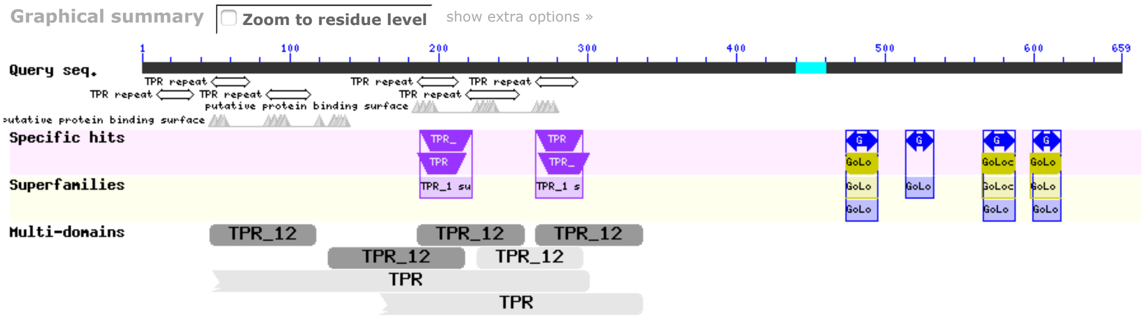

Pencil urchin

EtAGS(pencil urchin)

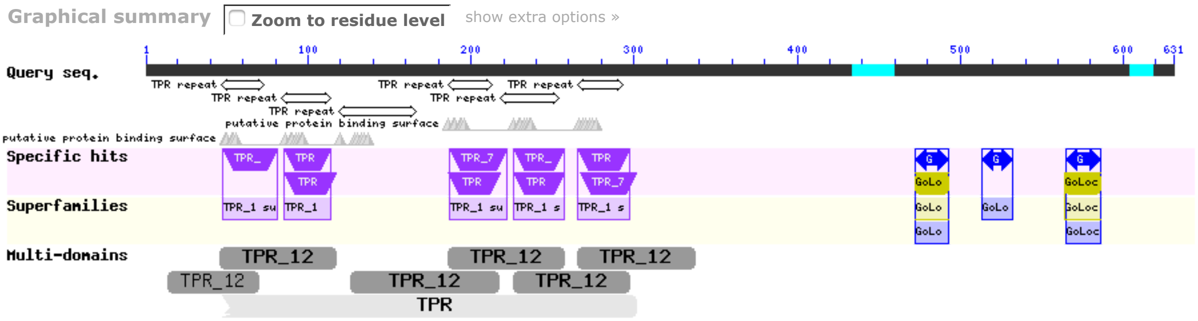

Sea Cucumber

SbAGS-Cucumber

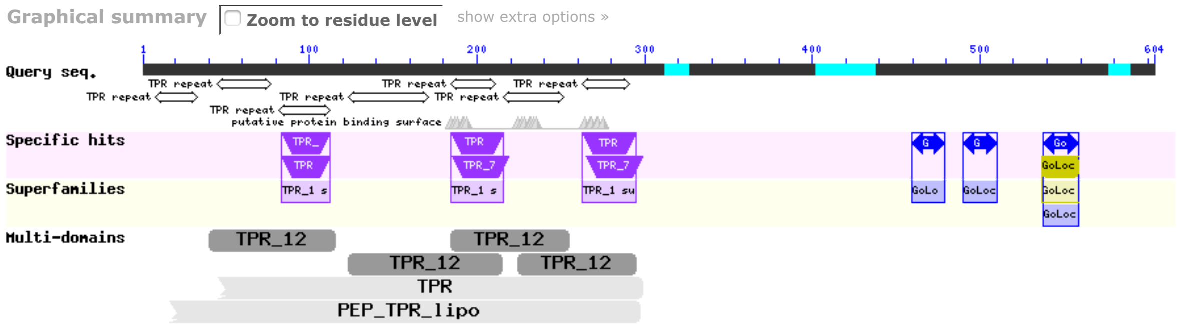

Sea star

PmAGS

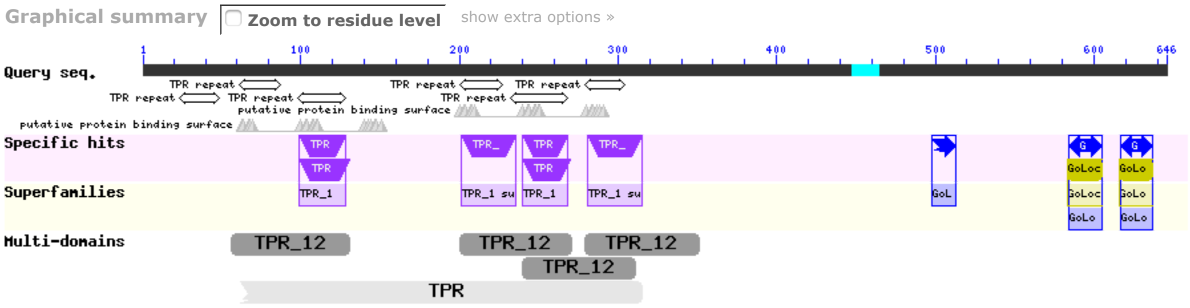

PpAGS(starfish)

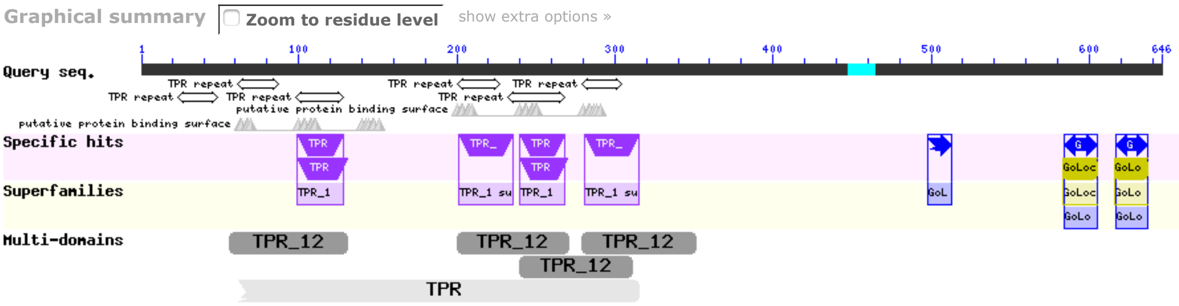

OjAGS (feather star)

OjAGS(feather star)

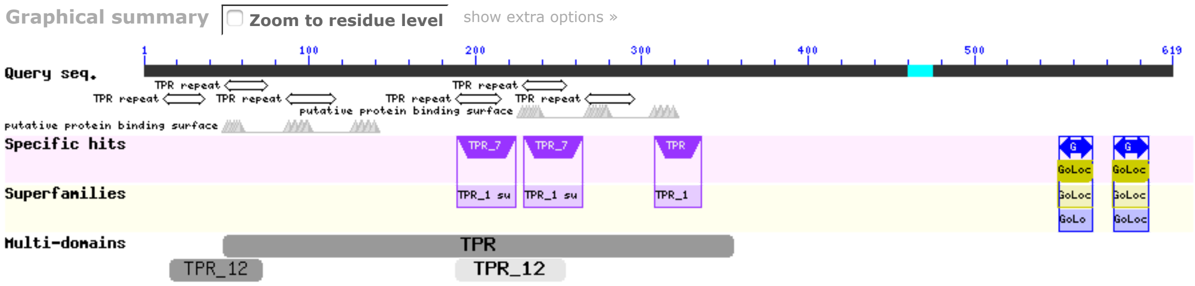

B. Sequences of GoLoco motif #1-4 of each echinoderm AGS

Ep (Sand dollar)
1: 412 DNFFEALSRFQSNRMDEQRCSF 433
3: 504 DDFFEMLMRCQGARIEDQRSSL 525
4: 537 EDFFSLIQRIQSKRIEEQR 555

Sp (Sea urchin)
1: 474 DNFFEALSRFQSNRMDEQRCSF 495
2p: 514 EELLDEIAKLQGSRLNEQRA 533
3: 566 DDFFEMIIRCQGARIEDQRSTL 587
4: 599 EDFFSLIQRIQSKRIEEQRS 618

Et (Pencil urchin)
1: 472 DNFFEALSRFQSNRMDEQRCSF 493
2p: 513 EELLDEIAKLQGSRLNEQRA 532
3: 565 DDFFEMLMRCQGARIEDQRSTL 586

Sb (Sea cucumber)
1p: 459 DFFDALSRFQEKRINDQRVSF 479
2p: 490 EDLLDEIAKVQGNRLNEQRAN 510
3: 538 DDFFEMLMRCQGARMEDQRSSL 559

Pm (Sea star)
1p: 498 LSRFQGKRMDEQRCSF 513
3: 584 DDFFDMLMRCQGSRINDQRSEP 605
4: 617 EDFFALIQRVQSKRMDAQRSD 637

Oj (Feather star)
3: 551 DDFFDMLIRCQGTRIEDQRTD 571
4: 584 EDFFSLISRVQSQRLEEQRCDL 605
**p indicates partial sequence*

C. Sequence comparison of GoLoco motif #1 among Fly Pins, human LGN, and sea urchin AGS

Fly Pins : DDFFEMLSRSQSKRMDDQRCS
Human LGN: EGFFDLL SRFQSNRMDDQRCC
Urchin AGS: DNFFEAL SRFQSNRMDEQRCS
Similarity: : . * * * * * * * * * : * * * : * * * .

* The conserved sequences are highlighted in red and the similar sequences are in orange.

Supplementary Table 8. A key resource table

REAGENT or RESOURCE	SOURCE	IDENTIFIER
**Antibodies**		
Anti-SpAGS	Ref # 10	N/A
Anti-Gαi	Santa Cruz Biotech	# sc-56536
Anti-suNuMA	Genescript	This article; Custom-made peptide antibody
Anti-PLK1 (rabbit polyclonal)	Abcam	# ab137352
Anti-PLK1 (mouse monoclonal)	Abcam	# ab14210
Anti-Endo1	Ref # 26	N/A
Anti-p150	BD Transduction Laboratories	# 610473
Anti-Dynein antibody	Millipore	# MAB1618
Anti-Phosphoserine antibody	Sigma-Aldrich	# P3430
Anti-beta-tubulin	Sigma-Aldrich	# F2043
Anti-YP30 antibody	Wessel et al, 2000	N/A
Cy3-conjugated goat anti-rabbit IgG	Thermo Fisher Scientific	# A10520
Alexa 488-conjugated goat anti-mouse IgG	Thermo Fisher Scientific	# A32723
Alexa 594-conjugated goat anti-mouse IgG	Thermo Fisher Scientific	# A11032
HRP-conjugated anti-Protein A antibody	Abcam	# ab7245
HRP-conjugated goat anti-rabbit IgG	Thermo Fisher Scientific	# A16096
HRP-conjugated goat anti-mouse IgG	Thermo Fisher Scientific	# 31430
**Chemicals, Peptides, and Recombinant Proteins**		
Hoechst 33342	Thermo Fisher Scientific	# 62249
PTX	Sigma-Aldrich	# P2980
SDS	Sigma-Aldrich	# L3771
Taxol (Paclitaxel)	Sigma-Aldrich	# T7402
Nocodazole	Sigma-Aldrich	# M1404
Ciliobrevin A	TOCRIS bioscience	# 302803-72-1
BI2536	Selleckchem	# S1109
Rhodamine-conjugated Phalloidin	Thermo Fisher Scientific	# R415
RITC	Sigma-Aldrich	# 283924
Red fluorescent dextran (Fluoro-Ruby)	Invitrogen	# D1817
PLK1 Recombinant Human Protein	Thermo Fisher Scientific	# PV3501
**Critical Commercial Assays**		
mMESSAGE mMACHINE SP6 Transcription Kit	Ambion	#AM1340
DIG RNA Labeling Kit (T7)	Roche	# 11 175 025 910
In-Fusion HD Cloning	Clonetech	# 639648
Dynabeads Protein A	Thermo Fisher Scientific	# 10001D
**Recombinant DNA**		
Plasmid: PLK1-GFP	Ref # 20	N/A
Plasmid: membrane-mCherry-PLK1	Ref # 20	N/A
Plasmid: membrane-mCherry-PLK1-dead	Ref # 20	N/A
Plasmid: Human AGS-GFP	Missouri S&T cDNA resource center	# AGS03L0000
Plasmid: Human LGN-GFP	Ref # 20	N/A
Plasmids: SpAGS-GFP, SpAGS-dC-term-GFP, SpAGS-dGoLoco1-GFP, Kaede-SpAGS	This article	N/A
Plasmid: GFP-SpGαi	This article	N/A
Plasmid: Vasa-GFP	# 5	N/A
Plasmid: mCherry-EMTB	Addgene	# 26742
**Software and Algorithms**		
Echinoderm protein sequences	EchinoBase	http://www.echinobase.org/Echinobase/
Protein motif search	NCBI blast	https://blast.ncbi.nlm.nih.gov/Blast.cgi
Protein sequence alignment	Clustal Omega	http://www.ebi.ac.uk/Tools/msa/clustalo/

**Primers**		
SPU #	*Name*	*Sequence*
SPU_009218 [http://www.echinobase.org/Echinobase/Search/SpSearch/viewAnnoGeneInfo.php?spu_id=SPU_009218]	Sp-Ags	F:ATGCAGAGCGAGGCCTCCTGTATGGAGC<br>R:TCAAAGGTGAGATTCTTGAACAGGTCTG
SPU_013414 [http://www.echinobase.org/Echinobase/Search/SpSearch/viewAnnoGeneInfo.php?spu_id=SPU_013414]	Sp-Gαi	F: ATGGGGTGCGCTACGAGCGCAGAAG<br>R: TTATTTTTTATTTTTTTTGGGGGGGGG

**Morpholino antisense oligos** (*Gene Tools: http://www.gene-tools.com/)*		
SPU #	*Name*	*Sequence*
SPU_009218 [http://www.echinobase.org/Echinobase/Search/SpSearch/viewAnnoGeneInfo.php?spu_id=SPU_009218]	Sp-Ags	GGCCCGTTTCACAAAGCCTTTGTTT
SPU_013414 [http://www.echinobase.org/Echinobase/Search/SpSearch/viewAnnoGeneInfo.php?spu_id=SPU_013414]	Sp-Gαi	GCTCGTAGCGCACCCCATGTTGGAA
SPU_023686 [http://www.echinobase.org/Echinobase/Search/SpSearch/viewAnnoGeneInfo.php?spu_id=SPU_023686]	Sp-NuMA (eIF3s5L)	CCTGCTCCAACTCCCTCATCCTTGC
SPU_017949 [http://www.echinobase.org/Echinobase/Search/SpSearch/viewAnnoGeneInfo.php?spu_id=SPU_017949]	Sp-PLK1	TTATGTCTTGAAGCCATCATCTTGC
SPU_025646 [http://www.echinobase.org/Echinobase/Search/SpSearch/viewAnnoGeneInfo.php?spu_id=SPU_025646]	Sp-Dsh	AATCTTAGTCTCCTCCATTATTGGA

Supplementary References

1. Hay, B., Jan, L. Y. & Jan, Y. N. A protein component of Drosophila polar granules is encoded by vasa and has extensive sequence similarity to ATP-dependent helicases. Cell 55, 577-587 (1988).
2. Lasko, P. F. & Ashburner, M. The product of the Drosophila gene vasa is very similar to eukaryotic initiation factor-4A. Nature 335, 611–617 (1988).
3. Carrera, P., Johnstone, O., Nakamura, A., Casanova, J., Jäckle, H. & Lasko, P. VASA mediates translation through interaction with a Drosophila yIF2 homolog. Mol. Cell 5, 181-187 (2000).
4. Sengoku, T., Nureki, O., Nakamura, A., Kobayashi, S. & Yokoyama, S. Structural basis for RNA unwinding by the DEAD-box protein Drosophila Vasa. Cell 125, 287-300 (2006).
5. Yajima, M. & Wessel, G. M. The DEAD-box RNA helicase Vasa functions in embryonic mitotic progression in the sea urchin. Development 138, 2217-2222 (2011).
6. Yajima, M. & Wessel, G. M. The germ line factor Vasa functions broadly in somatic cells: mRNA clustering, translational regulation, and wound healing. Development 142, 1960-1970 (2015).
7. Lu, M. S. & Johnston, C. A. Molecular pathways regulating mitotic spindle orientation in animal cells. Development 140,1843-56 (2013).
8. Takesono, A., Cismowski, M. J., Ribas, C., Bernard, M., Chung, P., Hazard, S. 3rd, Duzic, E. & Lanier, S. M. Receptor-independent activators of heterotrimeric G-protein signaling pathways. J. Biol. Chem. 274, 33202-33205. (1999).
9. Voronina, E. & Wessel, G. M. Regulatory contribution of heterotrimeric G-proteins to oocyte maturation in the sea urchin. Mech. Dev. 121, 247-259 (2004).
10. Voronina, E. & Wessel, G. M. Activator of G-protein signaling in asymmetric cell divisions of the sea urchin embryo. Dev, Growth Differ. 48, 549-557 (2006).
11. Morin, X. & Bellaı¨che, Y. Mitotic Spindle Orientation in Asymmetric and Symmetric Cell Divisions during Animal Development. Dev. Cell. 21, 102-119 (2011).
12. Du, Q., Stukenberg, P. T. & Macara, I. G. A mammalian Partner of inscuteable binds NuMA and regulates mitotic spindle organization. Nat. Cell Biol. 3, 1069-1075 (2001).
13. Yasumi, M., Sakisaka, T., Hoshino, T., Kimura, T., Sakamoto, Y., Yamanaka, T., Ohno, S. & Takai, Y. Direct binding of Lgl2 to LGN during mitosis and its requirement for normal cell division. J. Biol. Chem. 280, 6761-6765 (2005).
14. Fuja, T. J., Schwartz, P. H., Darcy, D. & Bryant, P. J. Asymmetric localization of LGN but not AGS3, two homologs of Drosophila pins, in dividing human neural progenitor cells. J. Neurosci. Res. 75, 782-793 (2004).
15. Yang, C. H., Lambie, E. J. & Snyder, M. NuMA: an unusually long coiled-coil related protein in the mammalian nucleus. J. Cell Biol. 116, 1303-1317 (1992).
16. Gueth-Hallonet, C., Weber, K. & Osborn, M. NuMA: a bipartite nuclear location signal and other functional properties of the tail domain. Exp. Cell Res. 225, 207-218 (1996).
17. Radulescu, A. E. & Cleveland, D. W. NuMA after 30 years: the Matrix Revisited. Trends. Cell Biol. 20, 214–222 (2010).
18. Haren, L. & Merdes, A. Direct binding of NuMA to tubulin is mediated by a novel sequence motif in the tail domain that bundles and stabilizes microtubules. J. Cell Sci. 115(Pt 9),1815-1824 (2002).
19. Du, Q., Taylor, L., Compton, D. A. & Macara, I. G. LGN blocks the ability of NuMA to bind and stabilize microtubules. A mechanism for mitotic spindle assembly regulation. Curr. Biol. 12, 19281933 (2002).
20. Kiyomitsu, T. & Cheeseman, I. M. Chromosome- and spindle-pole-derived signals generate an intrinsic code for spindle position and orientation. Nat. Cell Biol. 14, 311-317 (2012).
21. Sunkel, C. E. & Glover, D. M. polo, a mitotic mutant of Drosophila displaying abnormal spindle poles. J. Cell Sci. 89 (Pt 1), 25-38 (1988).
22. Llamazares, S., Moreira, A., Tavares, A., Girdham, C., Spruce, B. A., Gonzalez, C., Karess, R. E., Glover, D. M. & Sunkel, C. E. polo encodes a protein kinase homolog required for mitosis in Drosophila. Genes Dev. 5, 2153-2165 (1991).
24. Lee, K. S, Grenfell, T. Z., Yarm, F. R. & Erikson, R. L. Mutation of the polo-box disrupts localization and mitotic functions of the mammalian polo kinase Plk. Proc. Natl. Acad. Sci. U. S. A. 95, 9301-9306 (1998).
25. Takai, N., Hamanaka, R., Yoshimatsu. J. & Miyakawa, I. Polo-like kinases (Plks) and cancer. Oncogene 24, 287-291 (2005).
26. Wessel, G. M. & McClay, D. R. Sequential expression of germ-layer specific molecules in the sea urchin embryo. Dev. Biol. 111, 451-463 (1985).
