# Supplementary material for: Evolutionary modification of AGS protein contributes to formation of micromeres in sea urchins
Source: Nat Commun. 2019 Aug 22;10:3779. doi: 10.1038/s41467-019-11560-8 (PMC6706577; doi:10.1038/s41467-019-11560-8)
Supplement: Supplementary file 4 — Reporting Summary [file 41467_2019_11560_MOESM4_ESM.pdf]

nature research

Corresponding author(s): Mamiko Yajima

Last updated by author(s): 7/14/2019

Reporting Summary

Nature Research wishes to improve the reproducibility of the work that we publish. This form provides structure for consistency and transparency in reporting. For further information on Nature Research policies, see Authors & Referees and the Editorial Policy Checklist.

Statistics

For all statistical analyses, confirm that the following items are present in the figure legend, table legend, main text, or Methods section.

n/a	Confirmed	
☐	☒	The exact sample size (*n*) for each experimental group/condition, given as a discrete number and unit of measurement
☐	☒	A statement on whether measurements were taken from distinct samples or whether the same sample was measured repeatedly
☐	☒	The statistical test(s) used AND whether they are one- or two-sided<br>*Only common tests should be described solely by name; describe more complex techniques in the Methods section.*
☐	☒	A description of all covariates tested
☐	☒	A description of any assumptions or corrections, such as tests of normality and adjustment for multiple comparisons
☐	☒	A full description of the statistical parameters including central tendency (e.g. means) or other basic estimates (e.g. regression coefficient) AND variation (e.g. standard deviation) or associated estimates of uncertainty (e.g. confidence intervals)
☐	☒	For null hypothesis testing, the test statistic (e.g. *F*, *t*, *r*) with confidence intervals, effect sizes, degrees of freedom and *P* value noted<br>*Give P values as exact values whenever suitable.*
☒	☐	For Bayesian analysis, information on the choice of priors and Markov chain Monte Carlo settings
☒	☐	For hierarchical and complex designs, identification of the appropriate level for tests and full reporting of outcomes
☒	☐	Estimates of effect sizes (e.g. Cohen's *d*, Pearson's *r*), indicating how they were calculated

Our web collection on statistics for biologists contains articles on many of the points above.

Software and code

Policy information about availability of computer code

Data collection	Echinobase.org for collection of gene/protein information of echinoderms. NCBI blast site for collection of human gene/protein sequences. Confocal microscope with dedicated softwares (Olympus FV3000, Zeiss 800); Epifluorescent scope with Metaphopho software
Data analysis	Image J

For manuscripts utilizing custom algorithms or software that are central to the research but not yet described in published literature, software must be made available to editors/reviewers. We strongly encourage code deposition in a community repository (e.g. GitHub). See the Nature Research guidelines for submitting code & software for further information.

Data

Policy information about availability of data

All manuscripts must include a data availability statement. This statement should provide the following information, where applicable:

- Accession codes, unique identifiers, or web links for publicly available datasets
- A list of figures that have associated raw data
- A description of any restrictions on data availability

• The datasets generated during and/or analysed during the current study are available from the corresponding author on reasonable request.

Field-specific reporting

Please select the one below that is the best fit for your research. If you are not sure, read the appropriate sections before making your selection.

☒ Life sciences ☐ Behavioural & social sciences ☐ Ecological, evolutionary & environmental sciences

For a reference copy of the document with all sections, see nature.com/documents/nr-reporting-summary-flat.pdf

Life sciences study design

All studies must disclose on these points even when the disclosure is negative.

Sample size	Sample size of n=10 or larger was used for all experiments to test reproducibility of experiments, except for a special instances where the large number of sample preparation was technically difficult.
Data exclusions	Batches of animals that showed abnormal development at more than 50% in normal/non-manipulated embryogenesis were excluded.
Replication	At least three independent cycles of expriments were conducted for each experiment.
Randomization	For each set up experiment, the random animals (echinoderms) obtained from the wild ocean were used. No genetic line is available for these animals and thus no selection was applicable.
Blinding	Phenotype assays require trained eyes and thus were done by the multiple trained researchers. Multiple different experiments/approaches were also used to make a single conclusion to increase confidence in our results.

Reporting for specific materials, systems and methods

We require information from authors about some types of materials, experimental systems and methods used in many studies. Here, indicate whether each material, system or method listed is relevant to your study. If you are not sure if a list item applies to your research, read the appropriate section before selecting a response.

Materials & experimental systems

n/a	Involved in the study	
☐	☒	Antibodies
☒	☐	Eukaryotic cell lines
☒	☐	Palaeontology
☐	☒	Animals and other organisms
☒	☐	Human research participants
☒	☐	Clinical data

Methods

n/a	Involved in the study	
☒	☐	ChIP-seq
☒	☐	Flow cytometry
☒	☐	MRI-based neuroimaging

Antibodies

Antibodies used	See methods section for details.
Validation	Validation of each antibody was either done in the current work by immunoblot/immunofluorescence or in the previous studies (see citations in the text).

Animals and other organisms

Policy information about studies involving animals; ARRIVE guidelines recommended for reporting animal research

Laboratory animals	Sea urchin (S. purpuratus), Pencil urchin (E. tribuloides), Sea star (P. miniata); both males and females were used; all wild type obtained from the ocean.
Wild animals	Echinoderms were obtained by the vendor (see details in the methods section). No regulation is applicable to echinoderms and animals were processed for regular trashing procedure.
Field-collected samples	See above section
Ethics oversight	See above section

Note that full information on the approval of the study protocol must also be provided in the manuscript.
