# Supplementary material for: Evolutionary modification of AGS protein contributes to formation of micromeres in sea urchins
Source: Nat Commun. 2019 Aug 22;10:3779. doi: 10.1038/s41467-019-11560-8 (PMC6706577; doi:10.1038/s41467-019-11560-8)
Supplement: Supplementary file 5 — Description of Additional Supplementary Files [file 41467_2019_11560_MOESM5_ESM.docx]

**Title: Supplemental movie 1,** relevant to Fig. 5

**Description:** Overexpression of AGS protein (green) causes more symmetric division of the vegetal blastomeres during the 8-16 cell stage. Images taken every 15 seconds for 10 minutes.
